# "May I Buy a Pack of Marlboros, Please?" A Systematic Review of Evidence to Improve the Validity and Impact of Youth Undercover Buy Inspections

**DOI:** 10.1371/journal.pone.0153152

**Published:** 2016-04-06

**Authors:** Joseph G. L. Lee, Kyle R. Gregory, Hannah M. Baker, Leah M. Ranney, Adam O. Goldstein

**Affiliations:** 1 Department of Health Education and Promotion, College of Health and Human Performance, East Carolina University, Greenville, North Carolina, United States of America; 2 Tobacco Center of Regulatory Science, School of Public Health, Georgia State University, Atlanta, Georgia, United States of America; 3 Tobacco Prevention and Evaluation Program, Department of Family Medicine, School of Medicine, University of North Carolina at Chapel Hill, Chapel Hill, North Carolina, United States of America; 4 Lineberger Comprehensive Cancer Center, School of Medicine, University of North Carolina at Chapel Hill, Chapel Hill, North Carolina, United States of America; University College London, UNITED KINGDOM

## Abstract

Most smokers become addicted to tobacco products before they are legally able to purchase these products. We systematically reviewed the literature on protocols to assess underage purchase and their ecological validity. We conducted a systematic search in May 2015 in PubMed and PsycINFO. We independently screened records for inclusion. We conducted a narrative review and examined implications of two types of legal authority for protocols that govern underage buy enforcement in the United States: criminal (state-level laws prohibiting sales to youth) and administrative (federal regulations prohibiting sales to youth). Ten studies experimentally assessed underage buy protocols and 44 studies assessed the association between youth characteristics and tobacco sales. Protocols that mimicked real-world youth behaviors were consistently associated with substantially greater likelihood of a sale to a youth. Many of the tested protocols appear to be designed for compliance with criminal law rather than administrative enforcement in ways that limited ecological validity. This may be due to concerns about entrapment. For administrative enforcement in particular, entrapment may be less of an issue than commonly thought. Commonly used underage buy protocols poorly represent the reality of youths' access to tobacco from retailers. Compliance check programs should allow youth to present themselves naturally and attempt to match the community’s demographic makeup.

## Introduction

Tobacco products remain one of the most deadly consumer products, killing almost half of their users when used as directed [1]. Most smokers become addicted to tobacco products before they are legally able to purchase these products [2]. Stores that sell tobacco products ("tobacco retailers") play an important role in minors' access to tobacco products, and researchers and policymakers have long been interested in reducing the availability of tobacco products to youth [3].

Although 180 countries [4] are parties to the World Health Organization Framework Convention on Tobacco Control, of which Article 16 requires a prohibition of sales of tobacco products to youth, much less is known about how to implement bans on youth sales [5]. The most frequently employed method to assess sales to youth is an underage buy inspection, where a minor attempts to purchase tobacco products under the supervision of adults. Research in the 1980s and 1990s related to underage buys showed widespread access to tobacco products and conflicting evidence on the impact of restricting youth access on reducing youth tobacco use [6]. One explanation [7] of the conflicting findings has been a lack of validity in how youth access is measured while conducting underage buys [8]. That is, the tightly controlled, standardized, and scripted procedures implemented with well-dressed, non-smoking minors may not accurately reflect real-world youth access to tobacco products [8]. Ideal retailer compliance of 90% or higher in a given community may not be correctly measured, thereby limiting interpretation of the evidence of impact [7]. In the United States, both states and the federal government are investing heavily in underage buy inspections; the Food and Drug Administration (FDA), for example, has allocated over $178 million as of September 2015 and conducted over 495,000 retailer inspections of all types through state contractors since 2010 [9, 10].

States have been implementing underage buy inspections since 1992 under the Alcohol, Drug Abuse, and Mental Health Administration Reorganization Act [11], commonly called the Synar Amendment. The Synar Amendment required states to pass legislation prohibiting sales of tobacco products to minors and maintain a rate of retailer sales to minors under 20% or face substantial penalties to federal substance abuse block grant funding [11]. Although published retailer violation rates have decreased over time, Synar implementation has been mixed and funding dedicated to program implementation lacking [12, 13]. It is important to note that the Synar Amendment required enforcement of *state* youth access laws. That is, though enforcement was required by federal law, enforcement operated under youth access laws passed by the individual state. These laws generally prohibit the sale of tobacco to minors as a *criminal* offense. Under criminal law, a state proscribes a certain activity by making its conduct punishable and the state prosecutes violations of the law in a criminal court.

The Family Smoking Prevention and Tobacco Control Act of 2009 (FSPTCA) granted the FDA the authority to regulate tobacco products and implement regulations governing the sale and distribution of tobacco, including prohibiting the sale of cigarettes, cigarette tobacco, and smokeless tobacco to persons younger than 18. Unlike state criminal youth access laws, inspections conducted for the FDA under the FSPTCA seek to ensure compliance with an *administrative* regulation. Administrative regulations are designed as a tool of deterrence and compliance rather than punishment because they cannot result in imprisonment as punishment for a crime. Under the FSPTCA, FDA violations of selling tobacco products to minors result in a warning letter, civil financial penalty, or a no tobacco sales order (21 CFR 17). These violations are adjudicated through administrative hearings rather than criminal courts and are subject to lower evidence requirements than if they were settled in criminal courts. Moreover, the FDA regulations governing the sale of tobacco products operate on a different legal standard than criminal law. Liability arises simply through the existence of the minor sale violation. Intent is not taken into consideration and the FDA does not need to prove that the retailer knew the minor was underage (21 C.F.R. § 1140.14).

Although some previous research has examined characteristics of underage buyers and violation rates [14], best practices in youth access reduction programs [15], and implementation of underage buy programs [13], the literature examining characteristics of underage buy *protocols* remains un-synthesized. Nor has the literature on underage buy protocols been examined in relation to differences in criminal and administrative enforcement authority. Protocols that do not match the relevant enforcement authority may limit the impact of underage buy inspections programs. Synthesis of this literature should inform FDA, Synar, and other state or local efforts to develop and implement underage buy inspection protocols that maximize impact on youth access, including future efforts to address e-cigarette sales to minors.

The purpose of this research is to: (1) systematically examine the existing peer-reviewed evidence assessing underage buy protocols; (2) examine correlates of sales to minors in underage buy inspections that can be changed by state program staff, i.e., minor characteristics (age, race, gender) and neighborhood characteristics of the tobacco retailers that are chosen to be inspected; and (3) review the findings in relation to issues of entrapment with criminal vs. administrative enforcement authority.

## Methods

Our protocol is available as S1 File. We first developed search keywords in four domains (1) tobacco, (2) youth, (3) access, and (4) compliance check characteristics. We based initial keywords off a Cochrane Review [16] and iteratively developed keywords and controlled vocabulary until no new relevant results were found in PubMed. A librarian reviewed and enhanced our search strategy. Once we established a final search string in PubMed, we translated the controlled vocabulary (i.e., the indexing terms used in the database, MeSH terms in PubMed) into the controlled vocabulary of PsycINFO. We implemented our search on May 22, 2015, in PubMed and PsycINFO (the final search strings are available in S1 File). Because the first four volumes of the journal *Tobacco Control* are not indexed in PubMed [17], one author (KRG) searched these by hand. During the full-text data abstraction process, one author (JGLL) reviewed references in the introduction and discussion section of each included paper for additional relevant records.

**Fig 1:**
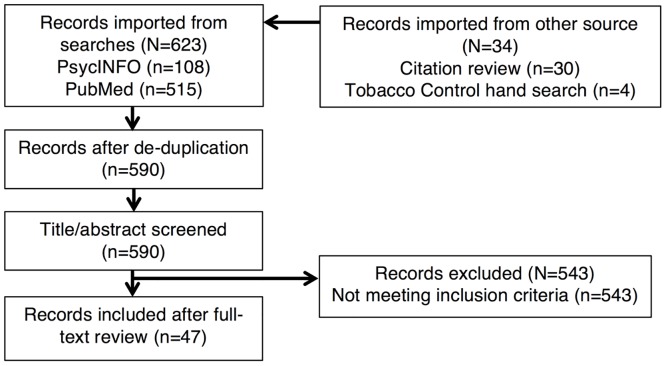
Inclusion flow diagram, May 22, 2015.

Two authors (JGLL and HMB) independently screened identified records' titles and abstracts for inclusion or exclusion. Disagreements were resolved by discussion with a third author (LMR) and by review of the full-text of the record. Fig 1 shows the inclusion process based on a PRISMA flow diagram [18]. We included records if they were published in peer-reviewed journals or as dissertations; addressed access to tobacco products by people under the age of 18; assessed underage purchase attempts or examined variations in implementation of purchase protocols or examined variations of purchase protocols; assessed the rate or likelihood of underage sales; were conducted from 1980 to the present; and were conducted in the United States. Conference presentations and published abstracts were not eligible for inclusion. We used no language restrictions. Records were classified as experimental or correlational based on the primary aims and methods of the record and reported in the respective evidence table. Once included, one author (JGLL) abstracted data directly into evidence tables in word processing software. For our second aim of assessing correlates of underage buy inspection non-compliance, we stratified results by our assessment of whether the study addressed confounding between minor age, race, gender, and area of assignment. One author coded confounding (JGLL) based on the methods used in each study, i.e., authors’ attempts to match youth in characteristics and equally assign youth with specific characteristics across neighborhood characteristics. Because our goal was to identify all existing studies, we conducted no further coding of risk of bias in included studies. We conducted a narrative review assessing the abstracted evidence, coming to a consensus about its implications for public health practice. One author, a lawyer with tobacco regulatory science training (KRG), then reviewed the findings and our conclusions against the legal authorities (i.e., criminal and administrative) that guide underage buy inspections programs with attention to the issue of entrapment. Regarding terminology, we use *youths* and *minors* interchangeably to indicate a person under 18 years of age.

## Results

We identified 47 records. Of these, ten studies experimentally assessed variations of underage buy protocols and 44 studies reported on the association of youth characteristics in underage buys and tobacco retailer non-compliance. We first discuss the experimental literature and then briefly summarize the evidence from correlational studies. Further details about correlational studies are presented in S1 Table.

Of the experimental research, studies mimicked youth behaviors [8] with assessments of familiarity (i.e., recognition by clerks as a returning customer) [19], truthfulness [8, 20, 21], use of minors who smoke [8], purchasing non-tobacco products (e.g., a bag of chips) during the attempt [20, 22], and using or not using an identification card [23, 24]. Additional experimental research examined the impact of non-cigarette tobacco purchases [25], assessments of more frequent inspections [26], and not completing purchases rung up by the store clerk [27]. Table 1 presents the details of these studies.

**Table 1:** Experimental Testing of Youth Tobacco Inspections Protocols, 1980–May 22, 2015, N = 10.

Setting: Design	Standard Protocol	Alternative	Results
**Cummings *et al*., 1996 [27]**			
Erie County, NY: Varied use of payment approach (youth paid for cigarettes) or termination approach (if clerk rang up sale, youth reported insufficient funds). Conducted in 1994 with 157 tobacco retailers. Same 16-year old boy used in all purchases. For both, when minor is asked age, he truthfully answered; when asked who cigarettes are for, he answered "me."	Enter store, pick up pack from display or ask clerk for Marlboros. (Purchasing pack is considered a successful sale.)	When pack is rung up by clerk, minor reports having inadequate funds. (Ringing up on the register is considered a successful sale.)	No difference between the two conditions, 61% payment, 68% no payment.
**DiFranza, Coleman, and St. Cyr, 1999 [25]**			
Worcester, MA: Varied product purchased using three 16-year old boys, who each made 34 purchase attempts with rotation in order of products purchased at stores and order in which boys purchased products for a total of 292 attempts. Each attempt made by different boy and separated by a few weeks. Conducted in 1998.	Non-smoking male youths entered store alone and attempted to purchase, provided no ID, and answered questions about age honestly.	Alternated product purchased between cigarettes, smokeless, and cigars.	No difference in sales by type of product purchased: 53.5% cigarettes, 55.6% for cigars, and 50.0% for cigars.
			"Although the three youths were all 15-year-old non-smokers, their individual sales rates varied from 26.5–88.4% (p < 0.001). The youths were successful in 45.5% of the first third of their attempts, in 53.6% of the middle third, and in 60.4% of the final third of their attempts to purchase tobacco. The trend of increasing success over time was significant (p < 0.05)" (p. 323).
**DiFranza, Savageau, and Bouchard, 2001 [8]**			
Eight communities, Massachusetts: Two attempts made on same day (within 30 minutes to standardize clerk) at 160 retailers, matched age, sex, and race/ethnicity. Order randomized.	Nonsmoking youth instructed to not wear heavy makeup or to appear older, stated true age, presented no ID; if refused, left quietly. Youth chooses brand. A bonus of $1 per completed sale was paid to the youth.	Young smokers natural behavior to buy tobacco in a new community: Current tobacco using youth allowed to dress as they chose, allowed to purchase other items while asking for cigarettes, allowed to lie about age, allowed to present own ID (not allowed to use fake ID). Could say anything to clerk except threats, expressions of anger, or profanity. Note only one girl had an ID. Youth chooses brand. Bonus of $1 per sale.	The odds of a sale were 5.7 times greater (95% CI: 1.5–22.0) in the alternative, more realistic protocol using smokers than in the standard protocol. This is controlling for sales clerk age, ID request, and community.
			"Smokers were almost twice as likely to be sold tobacco with no questions asked" (p. 230).
			Lying had little effect on results.
			ID effects may be confounded because only one youth had an ID.
**Hyland, Cummings, and Seiwell, 2000 [21]**			
Chautauqua County, NY: One white female, age 15, attempted 76 purchases in a single October 1998 weekend. Half of outlets in each city were randomized to each protocol. Adult remained outside and minor requested a pack of Marlboro cigarettes.	Truthfully reported age if asked, reported not having an ID.	Minor told merchant she was 18 if asked.	No difference in rate of sales by protocol, p = 0.48.
**Klonoff & Landrine, 2004 [20]**			
San Bernardino and Riverside County, CA: 1,600 purchase attempts in 232 stores with extensive training and seven waves of data collection, alternating standard protocol with alternative protocols to assess for secular trends. Each wave was separated by four to six weeks. Checks took place between 3 p.m. and 7 p.m. on weekdays and 9 a.m. and 4 p.m. on weekends. Racially/ethnically diverse participants, aged 15–17 (n = 21). All were non-smokers.	Standard protocol: "May I buy a pack of Marlboros, please?" If asked, cigarettes are for them, age is reported truthfully, and did not present ID.	Tested: (1) lie about age protocol, (2) note from dad protocol, (3) foot-in-door protocol. These are reported controlling for if the clerk requested ID. (1) "May I buy a pack of Marlboros, please? I'm 18." Youth repeatedly insisted being of age but would not produce an ID.	(1) Lying about age increased likelihood of sale, OR 4.22 (1.69–10.57)
		(2) "May I buy a pack of Marlboros, please? They're not for me, they're for my dad, here's a note from him." A fake note was presented.	(2) Note from dad decreased likelihood of sale, OR 0.13 (0.06–0.32)
		(3) Youth selected 2–3 items (e.g., soda/candy), placed on counter, waited until clerk began to ring up sale, and said "Oh, and a pack of Marlboros too please." Questions about age and who cigarettes were for were answered truthfully.	(3) Foot in the door purchase of sundries had no impact on sale, OR 0.88 (0.35–2.22).
			All manipulative conditions compared to standard, *x*^2^ = 4.408, df = 1, n = 1600, p < 0.04.
			Other than in the note from dad (which clerks "typically laughed at," p. 521) and rejected, requests for ID were lowest in the foot in the door protocol. Requesting ID was the strongest predictor of not selling.
**Landrine & Klonoff, 2003 [19]**			
San Bernardino and Riverside County, CA: Based on interviews with youth smokers, researchers designed an alternative protocol that was implemented by non-smoking youth age 15–17 in 232 randomly-selected tobacco retailers. Youth visited no more than seven retailers per week (one per day and one per clerk). Additional waves of data collection using the standard protocol (one prior, two after) were used to assess secular trends.	SAMHSA Synar Protocol: Enter store they are not known to and ask, "May I buy a pack of Marlboros, please?"	Familiarity protocol: Youths went to same store four times over six to eight days buying small items such as soda or candy from the same clerk and were friendly with the clerk. At visit five, youth then used standard protocol approach, walking up to register and asking, "May I buy a pack of Marlboros, please?"	Odds of a sale to a youth in the familiarity protocol were 5.51 (2.93–10.35) times greater than in the standard protocol.
			Authors note: "SAMSHA's prescribed method of sending youths into randomly selected stores where they are strangers must be modified to more accurately reflect youths' and merchants' behavior and hence actual youth access rates" (p. 1885).
**Landrine, Klonoff, *et al*., 2001 [24]**			
22 cities, California: Non-smoking minors age 15–17 attempted purchase at three time points to assess secular trends in 674 purchase attempts at 227 randomly selected retailers.	"May I have a pack of Marlboros, please?"	"May I have a pack of Marlboros please—I have ID" with flash of real California state ID or driver's license.	Odds of sale in the flash ID protocol were 3.8 time higher (2.05–7.03) than in the standard protocol.
**Levinson, Hendershott, and Byers, 2002 [23]**			
Six urban and suburban counties, Colorado: Non-smoking youths age 14–17 (n = 12, ten were male), most with previous compliance check experience, conducted 1,083 purchase attempts varying the presentation or non-presentation of a Colorado state ID (which notes the holder is under age 21). Males were clean shaven. Females wore no makeup. Adult supervisors were present in the store during the check.	If asked for ID, responded they didn't have one.	If asked for ID, provided actual ID.	When asked for ID, the ID condition had a relative risk 6.2 times more likelihood of sale than the no ID condition for a sales rate of: 12.2% (ID condition) vs. 1.9% (no ID condition). When standardized with covariates, RR 7.2 and sales rates of 12.6% v. 1.8%
			In final model with multiple covariates, odds of sale are 3.8 times greater for ID than no ID condition.
			Most (87.2%) clerks asked for an ID.
			"The current study suggests that the ID effect can increase cigarette sales to minors by nearly one third, even in locales where proof of age is usually required by clerks" (p. 298).
**Levinson & Patnaik, 2013 [26]**			
Three counties, Colorado: Using standard Synar protocol in one urban, one suburban, and one resort county, researchers conducted test-retest reliability assessment (1079 checks) in a census of 671 tobacco retailers. Minors (four female, seven male) were age 15.5–16, with A/B grades, non-smokers, clean shaven (males) with no tattoos, makeup, and modest/casual dress. Could enter alone or with another minor, answer questions evasively, buy snack or drink, use ID or not, and visit store several times before attempted purchase. Adults could stay outside or enter.	One check	Back-to-back checks separated by an average of 22.5 days. **Note**: Following classical test theory, unreliability in tests can be reduced by increasing the number of tests of the same construct	A single visit produced a retailer violation rate of 16.8% (first visit) or 15.7% (second visit); however, combining both visits produced a retailer violation rate of 25.3%.
			Purchase of a snack is associated with greater odds of sale, OR 2.50 (1.13–5.54)
			Odds of violation increased with minor experience.
**Schmidt, 2001 [22]**			
Baltimore, MD: Using a convenience sample of stores from a commercial list, conducted 237 checks. Three conditions to assess the role of purchase of an additional item with cigarettes.	Racially concordant pairs of girls, age 16.5–17.5, entered a store together. One of the pair attempted to purchase cigarettes in alternating fashion. All were non-smokers. White girls asked for "a pack of Marlboro Reds" and Black girls for "a pack of Newports." Answered questions about age truthfully, showed ID if asked.	Three conditions: (1) Purchase with adult item (i.e., newspaper), (2) purchase with small bag of potato chips, and (3) no co-purchase (i.e., control).	No difference in sale rate between three conditions, *x*^2^, df = 2, n = 237) = 0.213.

### Real-world youth behavior and familiarity in experimental studies

One of ten studies compared real-world youth behavior with a standard protocol [8]. Concerned about the validity of underage compliance checks, DiFranza and colleagues experimentally tested a protocol that used youth smokers, making no requirements on their dress, appearance, truthfulness (youth could say anything except threats, profanity, or expressions of anger), purchase of other items, or use of identification cards (but fake identification cards were not allowed) [8]. These youth smokers were compared against a standard protocol that used non-smoking youth who were clean-shaven and did not use makeup. The youth smokers had 5.7 times greater odds of being sold a tobacco product (95% CI: 1.5–22.0) after controlling for the sales clerk age, ID request, and community characteristics. The authors noted that youth smokers were nearly twice as likely to be sold a tobacco product as their non-smoking peers. One of the ten studies experimentally tested underage buy protocols with minors who became familiar with the clerk by visiting the store multiple times [19]. Landrine and Klonoff interviewed youth about how they attained access to tobacco products at retailers [19]. Because being acquainted with the retail clerk was one of the strongest themes, the authors designed an experiment to test familiarity. Familiarity with the minor (i.e., the minor had been in the store four previous times and interacted with the clerk but had not attempted a tobacco purchase over six to eight days previously) was associated with higher sales: the odds of a sale were 5.51 times greater (95% CI: 2.93–10.35) than in the standard protocol [19].

Other experimental studies examined specific aspects of real-world youth behaviors. Three studies examined the role of truthfulness in compliance rates [8, 20, 21]. Two studies reported on the use of actual IDs by youth during a compliance check [23, 24]. Two studies experimentally examined the role of purchasing additional items with a tobacco product [20, 22].

In two of three studies, youth lying about their age did not increase the likelihood of a tobacco sale [8, 21]. In the third, youth lying about their age increased the likelihood of the tobacco sale (OR = 4.22, 95% CI: 1.69–10.57) [20]. In this study, when the youth asked for a pack of Marlboros, they noted they were 18 and insisted on their age without providing ID [20]. In two studies, purchase of an additional item with the tobacco product did not change the likelihood of sale. In 2001, one study tested three buy conditions: purchase of tobacco items, purchase of a newspaper and tobacco, and purchase of a bag of chips and tobacco. There was no significant difference between the three conditions [22]. A second study from the early 2000s found no difference between a standard protocol and one in which, after several small items were purchased, the youth added, "Oh, and a pack of Marlboros too please" [20].

### Use of an identification card and type of product requested in experimental studies

One study compared youth flashing an ID quickly versus not using any ID [24]. Youth flashing of an ID resulted in 3.8 times greater likelihood of a sale (95% CI: 2.05–7.03) compared to no flashing of an ID [24]. In a separate study, if asked for an ID (which happened 87% of the time), the minor responded they had no ID or they provided an actual ID (which was marked "Under 21") [23]. When the youth presented the ID, there was a significantly higher rate of sales, relative risk was 6.2 times greater, than when no ID was presented [23].

One of the ten experimental studies addressed the relationship between tobacco product requested and likelihood of a sale [25]. In a 1999 experimental examination of purchases of cigarettes, smokeless tobacco, and cigars, no differences in sales were found [25]. Two studies were reported in three correlational records that included experimental manipulations that were not the primary purpose of the studies. In an experimental study that was published across two correlational records (showing results for pack purchases in one [28] and single cigarettes in the other [29]), youth were more likely to be sold the pack than the single cigarette [29]. In a 1995 study that used a partial split sample approach to select what product to buy, among boys (girls were not asked to purchase smokeless), the likelihood of sale of a smokeless product was greater than when a cigarette pack was requested [30].

### Purchase completion in experimental studies

One study looked at terminating the purchase after having the clerk ring up the sale, versus completing the purchase of the pack of cigarettes [27]. The youth would ask for a pack of cigarettes and then either pay for the cigarettes or make up an excuse about not having enough money to complete the purchase. In this study, not completing versus completing the purchase did not impact the calculation of the violation rate [27].

### Frequency of visits in experimental studies

One study examined the frequency of visits, comparing results between youth visiting stores a single time or visiting twice (an average of 23 days later) [26]. Visiting the same store twice (with different combinations of youth and clerks) produced a violation rate that was seven to eight percentage points higher than visiting just once. The authors conceptualized the study as a measurement study, showing limited reliability in a given underage buy attempt. Following Classical Test Theory, the addition of more checks of the same construct would increase measurement reliability, which is what the authors found [26].

### The role of minors’ experience

Although no studies experimentally examined youths’ experience directly, findings from one study that used youth smokers may be related to experience and comfort in purchasing cigarettes that would be more challenging for a non-smoking youth [8]. In ancillary analyses, a 1998 study found a significant dose-response relationship between youth experience with attempting to purchase tobacco products and their purchase rates, with rates of purchase in the last one-third of the project 15 percentage points higher than in the first one-third of the project [25].

### Youth and neighborhood characteristic correlates of underage sales

Of the 44 correlational records identified, we judged 20 to be at low risk of confounding between youth age, gender, race, and neighborhood characteristics. Details about each record are presented in S1 Table. Of these, 11 reported on age of youth [28, 29, 31–39], 13 on gender of youth [28–33, 35–41], six on race/ethnicity of youth [22, 28, 29, 37, 40, 41], and 14 on community characteristics associated with retailer sales rates [22, 28, 29, 35, 36, 39–47].

There is unequivocal evidence that the age of the minor attempting the purchase is positively associated with the likelihood of a sale. All 11 studies we identified as being at low risk of confounding reported a significant and positive association with age. The dose-response between these variables is striking: Authors in a 1996–1997 Massachusetts study reported a 4.2% buy rate for 13-year-old minors while 16-year-old minors had a 30.5% buy rate [34]. In a national study of FDA inspections, the odds of a tobacco sale to a 17-year-old minor were 2.43 times greater than for a 15-year-old minor [36].

The pattern of results suggests that girls are more likely to be sold tobacco products than boys. Indeed, of the studies at low risk of confounding, seven studies found positive associations between female gender and sales [28, 31–33, 36, 37, 39]. Five studies found no significant differences [29, 30, 38, 40, 41]. No studies found a positive association between male gender and likelihood of a sale. One study reported on gender discordance between the clerk and the buyer, finding higher rates of sales from female clerks to female minors and male minors than from male clerks to male minors [35].

#### Race and ethnicity

Of the six studies that reported on the role of the race or ethnicity of the youth and addressed confounding with other youth characteristics, five of the six showed significantly higher likelihood of sales to a Black youth than a White youth [22, 28, 37, 40, 41]. The remaining study, which examined only the sale of single cigarettes to youth, found no significant difference by the race of the minor [29].

#### Neighborhood characteristic correlates of underage sales

The literature on whether the likelihood of a sale differs by the demographic characteristics of a given neighborhood is complicated by a diversity of definitions of neighborhoods, limited reporting of unadjusted results, and the use of dichotomized neighborhood characteristics (which can reduce the power to detect an association [48]). Two records reported decreased likelihood of sales to minors as neighborhood income increased: Asumda and colleagues used *t*-tests to compare census block groups with a violation to all census block groups in Florida, finding lower per capita income in block groups with a violation [46]. Schmidt found similar results in Baltimore, MD [22]. However, these findings were not replicated in results adjusted for multiple covariates in Los Angeles, California, zip codes and Seattle, Washington, zip codes [35, 42] or in unadjusted results from a Midwestern city's census block groups comparing the top decile against all others [44].

Evidence of the relationship between the percentage of Black residents in a given neighborhood and the likelihood of a store's sale to minors was also complex. In adjusted regression analysis, positive associations were found in Los Angeles, CA, zip codes and Washington, DC, block groups. In unadjusted analyses, there was no significant relationship in four studies [28, 40, 41, 44] and a positive association in three studies [22, 29, 45], two of which were for the purchase of single cigarettes [29, 45]. Two studies found negative associations between Black residents and retailer sales to minors, one in Florida block groups [46] and the other in four San Diego, CA, communities as defined by the authors [39].

Regarding the proportion of Hispanic residents in a neighborhood and underage sales, four studies found significant positive associations in Florida block groups [46], San Diego, CA, communities defined by the authors, [39] a Midwestern metropolitan area’s block groups, [44] and, for single cigarettes, San Bernardino, CA, census tracts [29]. One study found no significant association for cigarette packs in San Bernardino, CA, census tracts [40].

### Legal considerations

Our review of the protocols in relation to criminal and administrative authority suggests the differences between the legal powers authorizing underage buy inspections matter. Public health professionals appeared to be concerned about underage purchase attempts constituting entrapment and designed restrictive, ecologically invalid protocols in response to this concern. The existing legal literature suggests this fear may be unsubstantiated. Entrapment would occur when compliance check personnel acquired the evidence necessary to prosecute a potential violator by inducing the retailer to violate the law when the retailer would not have otherwise done so. Simply put, if the intent originated in the mind of the inspection program rather than the retailer, the retailer has a potential defense. The issue of entrapment for retail compliance checks conducted under the FSPTCA has not been raised thus far by a retailer but such a defense is highly unlikely to succeed. To meet the legal standard for entrapment, significant inducement or coercion is required—something well beyond the straightforward underage tobacco purchase attempt—and it must be proven that the retailer would not have sold to the minor under regular (non-compliance check) circumstances. Historically, this has been extremely difficult to prove, particularly in situations involving the sale of alcohol and tobacco to minors (55 A.L.R.2d 1322). As a general rule, the use of underage buyers to expose illegal activity or regulatory violations does not constitute entrapment because the inspection does no more than offer the opportunity to commit the violation [49, 50].

## Discussion

Based on the existing experimental research, commonly used underage buy protocols appear to poorly represent the reality of youths' access to tobacco products from tobacco retailers for two reasons: (1) local youth making tobacco purchases are likely to be known by (i.e., become familiar to) clerks over time and (2) most youth tobacco purchasers look like, act like, and are (logically) tobacco users. In traditional measurement terminology, standard compliance checks have concerning issues with validity and additionally with reliability; a single compliance check is subject to considerable variability based on the age and experience of the minor conducting it as well as the clerk on duty [26]. Efforts are needed to reduce the use of younger rather than older minors in underage buy inspections and to maximize impact by targeting underage buy inspections where non-compliance is highest.

Using younger youth in underage buy protocols raises important issues regarding validity because younger youth are less likely to purchase tobacco from a retailer and more likely to get cigarettes from older acquaintances [2]. A previous review and General Accounting Office (GAO) report indicated older youth are more likely to be sold a tobacco product than younger youth at a tobacco retailer [13, 14]. Our review confirms that finding; there is striking evidence that older youths have much higher rates of purchase in underage buy inspections. The GAO recommended the removal of 15-year-olds from use in state inspections [13]. The use of 15-year-olds likely further reduces the validity of underage buy protocols. Use of 15-year-olds may assist in criminal prosecution of retailers in local courts by countering judicial views of underage tobacco sales being unimportant [51].

Programs implementing underage buy inspections under criminal statutory authority may need to be aware of local courts, public opinion, and pressure placed on health departments and prosecutors because these have proven to be barriers to underage buy programs [51, 52]. FDA administrative hearings are less likely to be susceptible to the pressures of local business interests. Administrative law judges are not elected by the community, nor do they work with local prosecutors or have relationships with local law enforcement.

There is some evidence for differences in likelihood of a sale by the demographic makeup of the neighborhood; sampling strategies for retail compliance checks should take into account potential disparities in violation rates and target areas with lower income. Evidence published after our search shows clear evidence of neighborhood disparities in the sale of single cigarettes by the racial and economic composition of neighborhoods [53].

We believe that the existing experimental research offers some clear improvements to the standard underage buy protocol (Table 2); whether or not they are for enforcement of criminal state laws or administrative FDA regulations should influence the protocols.

**Table 2:** Recommendations for Protocol.

**Recommendations for Youth Recruitment, Age, and Appearance**	**Rationale**
States should consider using minors no younger than 16, though exclusively using minors age 17 is ideal.	Younger minors often acquire their cigarettes from older youth and young adults.
States should strive for minors who represent real-world youth smokers, reflecting the gender and racial/ethnic composition of their locale.	It is illegal to sell to a 17-year-old regardless of appearance.
States should not artificially make minors look younger by requiring them to dress in a particular way (business casual dress codes, no makeup, no facial hair, etc.). For hiring purposes, states should consider interviewing minors by phone to avoid biasing appearance.	Efforts at using youthful appearing minors bias inspection results.
States should train and maintain experienced minors, including minor smokers who smoked before being recruited*.	(1) Real-world minor smokers are able to project confidence during a purchase attempt. (2) Using minors who smoke improves the validity of the purchase attempt, however programs leveraging minors who smoke have an ethical obligation to provide meaningful resources to quit.
**Recommendations for Protocol**	**Rationale**
States should vary the requested tobacco product to match the product typically purchased by minors of each particular demographic.	To reflect real-world minor behavior, products selected should match community and minor characteristics.
States should require minors to carry identification cards and show them *if asked*.	Presentation of ID does not necessarily preclude sale of tobacco products, and presentation of ID cards more closely reflects real-world experience of underage purchase attempts because some underage youth may assume that the clerk would not actually check the date of birth.
States should train minors in avoiding answering questions to disclose a compliance check, but there is no evidence to suggest lying about age improves validity of compliance checks.	Lying about age does not change the outcome of a purchase attempt. However, if retailers use answering this question as a way to identify a compliance check in progress, it may not be legally problematic to allow minors to lie.
States should consider sending the same minor to conduct purchase attempts more than once at the same store.	(1) Using a familiarity protocol clearly reflects real-world behaviors of neighborhood youth. (2) More frequent visits improve the reliability of underage buy rate measurement and, with enforcement, improve compliance. (3) More frequent visits improve the reliability of underage buy rate measurement and, with enforcement, improve compliance.

Note: Some state laws may preempt these recommendations and readers are advised to consult with local attorneys general to ensure compliance.

*States should make available smoking cessation resources to any employed minor smokers. Starting to smoke while participating in the program should be grounds for dismissal.

### Other considerations and future research

The literature identified does not address several important issues of underage buy compliance check protocols including how and when retailers become aware that they are being assessed for compliance. The use of adult chaperones who enter with the minor and the use of "unmarked" police cars (e.g., the classic gray sedans with tinted windows and extra antennas) may alert the retailer to a compliance check, again reducing the validity of the underage buy protocol. Further investigation of best practices to reduce inadvertent disclosure of a compliance check in process is needed. It is unclear if youth are more or less likely to be sold to depending on what brand they request. As the retail environment continues to evolve, protection of minors remains of paramount concern; at least one study shows no negative impacts from participation [54]. Additionally, with the FSPTCA and changes in legal landscape, there are new considerations for the age of the minor. We note that in FDA regulations, retailers should card all purchasers who appear to be under 28 years old. Failing to card a 19-year-old would still be a violation of the FSPTCA, although the sale itself would be legal (21 CFR 1140, FSPTCA Section 102). With growing adoption of Tobacco 21 laws increasing the legal age of sale to 21, young adults may be needed to participate in retailer inspection programs. Future research should assess how to optimize the ages of youth (or potentially young adult) inspections to maximize population health. Additionally, future research should (1) seek to disentangle the role of different real-world youth behaviors and (2) examine disparities in retailer sales to minors by neighborhood characteristics (e.g., income, racial composition) in wider geographic areas.

### Limitations

There are a number of limitations to this review. First, the age of the identified literature raises questions about (1) changes in the format of state-issued identification and (2) changes in the retail environment. The identified literature is older and thus may not reflect newer advances in vertical or color-coded licenses for youth under 18 or 21. However, it's unclear if use of an ID reflects real-world purchase attempts by youth [23]. The age of the existing literature is also a cause for concern given changes in the retail environment: Although self-service displays of tobacco products and vending machines were widely available in the 1990s, the contemporary retail landscape is substantially different. Second, due to an omission in our protocol and record-tracking system, we failed to record detailed information on the reasons records were excluded. Third, because of the limited literature and its age, we do not exclude records based on their quality or risk of bias instead opting to present as much of the existing evidence as we could identify. Future work should more thoroughly assess records for risk of bias. Like any systematic review, records not identified in our search may have been omitted. Publication bias may be present. Although our search reviewed citations in included records, we did not search for unpublished reports or government documents.

## Conclusions

The standard underage purchase protocol can be improved. State programs developing or updating protocols should consider the legal authority under which the protocol is being implemented and strive to mimic real-world appearance, behaviors, and demographics of participating youth in protocol development. Developing standard protocols that focus on the validity and reliability of the data is key to identifying violators, remedying the problem, and ultimately reducing youth access to tobacco products.

Previous federally mandated inspections programs have shown evidence of inconsistent implementation by states and used protocols that poorly reflect real-world levels of youth access to tobacco products at retailers [13, 15]. Current FDA inspections of tobacco retailers also show some evidence of differences in implementation by FDA’s state subcontractors in the identification of single cigarette sales [55]. Both states and researchers can improve the validity and reliability of underage buy protocols. As new efforts to reduce youth access to e-cigarettes emerge, there will be additional reasons for improving underage buy protocols. These improvements would likely help maximize the impact of scarce public health resources.

## Supporting Information

S1 File Protocol for Underage Buys: Validity and Implementation Systematic Review. (DOCX)

S2 File PRISMA 2009 Checklist. (DOC)

S1 Table Evidence Regarding Youth, Protocol, and Neighborhood Correlates of Non-Compliance with Prohibition on Sales of Tobacco Products to Minors, May 22, 2015, N = 44, Stratified by Risk of Confounding. (DOCX)
