# Supplementary material for: "May I Buy a Pack of Marlboros, Please?" A Systematic Review of Evidence to Improve the Validity and Impact of Youth Undercover Buy Inspections
Source: PLoS One. 2016 Apr 6;11(4):e0153152. doi: 10.1371/journal.pone.0153152 (PMC4822877; doi:10.1371/journal.pone.0153152)
Supplement: S1 File — (DOCX) [file pone.0153152.s001.docx]

**S1 File. Protocol for Underage Buys: Validity and Implementation Systematic Review**

Joseph G. L. Lee, PhD, MPH

Kyle R. Gregory, JD, MSHA

Hannah M. Baker, MPH

Leah M. Ranney, PhD

Adam O. Goldstein, MD, MPH

* Author order subject to change per ICMJE criteria.

East Carolina University

Georgia State University

Tobacco Prevention and Evaluation Program, Department of Family Medicine, School of Medicine, University of North Carolina at Chapel Hill

**Background**: The validity of youth access inspections (i.e., underage purchase attempts) of tobacco products has been widely questioned. That is, real-world access to tobacco products from retailers appears to be much greater for youth than is identified in standard compliance checks. As the FDA is contracting with states to conduct hundreds of thousands of these compliance checks under the FSPTCA of 2009, implementation of these checks by states should leverage existing research on increasing the validity of youth access inspections. Yet, the existing research on validity and implementation of youth access inspections is not synthesized, is scattered across multiple journals, and may not be readily available to state programs implementing FDA inspections. Additionally, some best practices from the research literature may not be allowed under the FSPTCA.

**Research Question**: What can states implementing FSPTCA underage buy inspections do to maximize their validity (and thus population impact)?

**Specific Aims**:

Aim_1_: Identify strategies that increase the validity of youth access inspections.

Aim_2_: Identify characteristics of the purchase attempt that are associated with the purchase attempt outcome.

Aim_3_: Identify characteristics of the youth that are associated with the purchase attempt outcome.

Aim_4_: Identify characteristics of the neighborhood that are associated with the purchase attempt outcome.

Aim_5_: Assess the evidence for unique characteristics of single cigarette purchase attempts.

Aim_6_: Assess the identified strategies and characteristics with an eye towards health equity, validity, and the legality of their implementation.

**PICOT (Population, Intervention, Comparator, Outcomes, Time, Setting)**:

- Population: Studies of youth (age < 18)
- Intervention: Youth access purchase attempts
- Comparator: Variations in implementation or purchase protocol
- Outcomes: Rate of underage sales
- Time: 1980 to present
- Setting: USA

Inclusion Criteria:

- U.S.-based study
- Any date
- Empirically assesses association between characteristic of youth, neighborhood or purchase attempt protocol on likelihood or validity of underage sale. (Retailer characteristics alone are not sufficient.)

Data Abstraction

- Coded by one author
- Create evidence table
  - Setting
  - Study Type, Year
    - Experiment with randomization
    - Correlation
  - Characteristics Assessed
  - Results
  - Recommendations
- Assessment of legal possibility

Search Strategy:

- Iterative search terminology developed
- Translation of controlled vocabulary
- Databases
  - PubMed
  - PsycINFO
- Hand search *Tobacco Control* (volumes 1 - 4)^1^
- Cross-check key references
- Independent inclusion/exclusion coding by two authors (JGLL, HMB)

Protocol

- This protocol will be updated with revisions and revisions will be dated.

| **Date, Notes**  **N** | **Tobacco** | **Access** | **Youth** | **Validity, Characteristics** |
| --- | --- | --- | --- | --- |
| 2015-05-01, Starting with Cochrane Review (Stead & Lancaster 2005) | Smoking cessation OR tobacco OR cigar* OR SMOKING-CESSATION OR TOBACCO-USE-DISORDER OR TOBACCO OR NICOTINE OR TOBACCO-SMOKELESS OR SMOKING/ prevention-and-control , therapy , legislation-and-jurisprudence OR ((quit* OR stop* OR ceas* OR giv*) near smoking) | sale OR sales OR retail OR retailer* OR store OR stores OR sell OR selling OR shop OR shops OR tobacconist* OR vending OR vendor* OR merchant* OR COMMERCE/ legislation-and-jurisprudence | adolescen* OR minors OR under()age OR student* OR (young near2 people) OR children OR juveniles OR girls OR boys OR teenager* OR teens OR child |  |
| 2015-05-01, Cleaning up to use TIAB, MeSH  2144 | Smoking cessation[MeSH] OR "smoking cessation"[tiab] OR tobacco[MeSH] OR tobacco[tiab] OR cigar* OR tobacco-use disorder[MeSH] OR tobacco products[MeSH] OR smoking[MeSH] OR smoking[tiab] OR ((quit* OR stop* OR ceas* OR giv*) near smoking) | sale[tiab] OR sales[tiab] OR retail[tiab] OR retailer*[tiab] OR store[tiab] OR stores[tiab] OR sell[tiab] OR selling[tiab] OR shop[tiab] OR shops[tiab] OR tobacconist*[tiab] OR vending[tiab] OR vendor*[tiab] OR merchant*[tiab] OR "Commerce/legislation and jurisprudence"[MeSH] OR access[tiab] | adolescen*[tiab] OR adolescent[MeSH] OR minors[tiab] OR minors[MeSH] OR underage[tiab] OR "under age"[tiab] OR student*[tiab] OR (young[tiab] near people[tiab]) OR children[tiab] OR juveniles[tiab] OR girls[tiab] OR boys[tiab] OR teenager*[tiab] OR teens[tiab] OR child[tiab] OR youth[tiab] OR youths[tiab] |  |
| 2015-05-01, expand search with other terminology from other reviews  2144 | Smoking cessation[MeSH] OR "smoking cessation"[tiab] OR tobacco[MeSH] OR tobacco[tiab] OR tobacco use[MeSH] OR cigar* OR tobacco-use disorder[MeSH] OR tobacco products[MeSH] OR (smoking[MeSH] NOT marijuana smoking[MeSH]) OR smoking[tiab] OR ((quit* OR stop* OR ceas* OR giv*) near smoking) OR cigarette[tiab] OR cigarettes[tiab] OR cigar[tiab] OR cigars[tiab] OR cigarillo[tiab] OR cigarillos[tiab] | sale[tiab] OR sales[tiab] OR retail[tiab] OR retailer[tiab] OR retailers[tiab] OR store[tiab] OR stores[tiab] OR sell[tiab] OR selling[tiab] OR shop[tiab] OR shops[tiab] OR tobacconist*[tiab] OR vending[tiab] OR vendor*[tiab] OR merchant*[tiab] OR "Commerce/legislation and jurisprudence"[MeSH] OR access[tiab] | adolescen*[tiab] OR adolescent[MeSH] OR minors[tiab] OR minors[MeSH] OR underage[tiab] OR "under age"[tiab] OR student*[tiab] OR (young[tiab] near people[tiab]) OR children[tiab] OR juveniles[tiab] OR girls[tiab] OR boys[tiab] OR teenager*[tiab] OR teens[tiab] OR child[tiab] OR youth[tiab] OR youths[tiab] |  |
| 2015-05-01, now let's see if we can get it down to characteristics and implementation + keyword of loosies  244 | Smoking cessation[MeSH] OR "smoking cessation"[tiab] OR tobacco[MeSH] OR tobacco[tiab] OR tobacco use[MeSH] OR cigar* OR tobacco-use disorder[MeSH] OR tobacco products[MeSH] OR (smoking[MeSH] NOT marijuana smoking[MeSH]) OR smoking[tiab] OR ((quit* OR stop* OR ceas* OR giv*) near smoking) OR cigarette[tiab] OR cigarettes[tiab] OR loosies[tiab] OR cigar[tiab] OR cigars[tiab] OR cigarillo[tiab] OR cigarillos[tiab] |  |  | discrimination[tiab] OR validity[tiab] OR reproducibility of results[MeSH] OR "neighborhood characteristics"[tiab] OR implementation[tiab] OR protocol[tiab] OR "best practices"[tiab] OR "best practice"[tiab] OR research design[MeSH] OR interpersonal relations[MeSH] |
| 2015-05-01, Let's take out cessation and see what that does. Makes not difference.  244 | tobacco[MeSH] OR tobacco[tiab] OR tobacco use[MeSH] OR tobacco-use disorder[MeSH] OR tobacco products[MeSH] OR (smoking[MeSH] NOT marijuana smoking[MeSH]) OR smoking[tiab] OR cigarette[tiab] OR cigarettes[tiab] OR loosies[tiab] OR cigar[tiab] OR cigars[tiab] OR cigarillo[tiab] OR cigarillos[tiab] |  |  |  |
| 2015-05-01, add in other discrimination, disparities measures  949 |  |  |  | discrimination[tiab] OR validity[tiab] OR valid[tiab] OR reproducibility of results[MeSH] OR implementation[tiab] OR protocol[tiab] OR protocols[tiab] OR "best practices"[tiab] OR "best practice"[tiab] OR research design[MeSH] OR interpersonal relations[MeSH] OR socioeconomic factors[MeSH] OR disparity[tiab] OR disparities[tiab] OR health status disparities[MeSH] OR inequality[tiab] OR inequalities[tiab] OR equity[tiab] OR inequity[tiab] OR inequities[tiab] OR neighbourhood[tiab] OR neighbourhoods[tiab] OR neighborhood[tiab] OR neighborhoods[tiab] OR residence characteristics[MeSH] OR "residence characteristics"[tiab] OR contrasting[tiab] OR community[tiab] OR communities[tiab] |
| 2015-05-01, let's take out community keywords -- they are too broad + add in availability as access synonym.  848 |  | sale[tiab] OR sales[tiab] OR retail[tiab] OR retailer[tiab] OR retailers[tiab] OR store[tiab] OR stores[tiab] OR sell[tiab] OR selling[tiab] OR shop[tiab] OR shops[tiab] OR tobacconist*[tiab] OR vending[tiab] OR vendor*[tiab] OR merchant*[tiab] OR "Commerce/legislation and jurisprudence"[MeSH] OR access[tiab] OR availability[tiab] |  | discrimination[tiab] OR validity[tiab] OR valid[tiab] OR reproducibility of results[MeSH] OR implementation[tiab] OR protocol[tiab] OR protocols[tiab] OR "best practices"[tiab] OR "best practice"[tiab] OR research design[MeSH] OR interpersonal relations[MeSH] OR socioeconomic factors[MeSH] OR disparity[tiab] OR disparities[tiab] OR health status disparities[MeSH] OR inequality[tiab] OR inequalities[tiab] OR equity[tiab] OR inequity[tiab] OR inequities[tiab] OR neighbourhood[tiab] OR neighbourhoods[tiab] OR neighborhood[tiab] OR neighborhoods[tiab] OR residence characteristics[MeSH] OR "residence characteristics"[tiab] OR contrasting[tiab] |
| 2015-05-01, let's try removing neighborhood keywords; eliminates relevant papers  828 |  |  |  | discrimination[tiab] OR validity[tiab] OR valid[tiab] OR reproducibility of results[MeSH] OR implementation[tiab] OR protocol[tiab] OR protocols[tiab] OR "best practices"[tiab] OR "best practice"[tiab] OR research design[MeSH] OR interpersonal relations[MeSH] OR socioeconomic factors[MeSH] OR disparity[tiab] OR disparities[tiab] OR health status disparities[MeSH] OR inequality[tiab] OR inequalities[tiab] OR equity[tiab] OR inequity[tiab] OR inequities[tiab] OR residence characteristics[MeSH] OR contrasting[tiab] |
| 2015-05-01, added in some neighborhood terms  838 |  |  |  | discrimination[tiab] OR validity[tiab] OR valid[tiab] OR reproducibility of results[MeSH] OR implementation[tiab] OR protocol[tiab] OR protocols[tiab] OR "best practices"[tiab] OR "best practice"[tiab] OR research design[MeSH] OR interpersonal relations[MeSH] OR socioeconomic factors[MeSH] OR disparity[tiab] OR disparities[tiab] OR health status disparities[MeSH] OR inequality[tiab] OR inequalities[tiab] OR equity[tiab] OR inequity[tiab] OR inequities[tiab] OR residence characteristics[MeSH] OR contrasting[tiab] OR "neighborhood"[tiab] OR "community characteristics" |
| 2015-05-01, do we need a Synar keyword and FDA[MeSH]? This seems to help somewhat.  881 |  |  |  | discrimination[tiab] OR validity[tiab] OR valid[tiab] OR reproducibility of results[MeSH] OR implementation[tiab] OR protocol[tiab] OR protocols[tiab] OR "best practices"[tiab] OR "best practice"[tiab] OR research design[MeSH] OR interpersonal relations[MeSH] OR socioeconomic factors[MeSH] OR disparity[tiab] OR disparities[tiab] OR health status disparities[MeSH] OR inequality[tiab] OR inequalities[tiab] OR equity[tiab] OR inequity[tiab] OR inequities[tiab] OR residence characteristics[MeSH] OR contrasting[tiab] OR "neighborhood"[tiab] OR "community characteristics"[tiab] OR Synar[tiab] OR United States Food and Drug Administration[MeSH] |
| 2015-05-08, Version sent to Lara Handler | tobacco[MeSH] OR tobacco[tiab] OR tobacco use[MeSH] OR tobacco-use disorder[MeSH] OR tobacco products[MeSH] OR (smoking[MeSH] NOT marijuana smoking[MeSH]) OR smoking[tiab] OR cigarette[tiab] OR cigarettes[tiab] OR loosies[tiab] OR cigar[tiab] OR cigars[tiab] OR cigarillo[tiab] OR cigarillos[tiab] | sale[tiab] OR sales[tiab] OR retail[tiab] OR retailer[tiab] OR retailers[tiab] OR store[tiab] OR stores[tiab] OR sell[tiab] OR selling[tiab] OR shop[tiab] OR shops[tiab] OR tobacconist*[tiab] OR vending[tiab] OR vendor*[tiab] OR merchant*[tiab] OR "Commerce/legislation and jurisprudence"[MeSH] OR access[tiab] OR availability[tiab] | adolescen*[tiab] OR adolescent[MeSH] OR minors[tiab] OR minors[MeSH] OR underage[tiab] OR "under age"[tiab] OR student*[tiab] OR (young[tiab] near people[tiab]) OR children[tiab] OR juveniles[tiab] OR girls[tiab] OR boys[tiab] OR teenager*[tiab] OR teens[tiab] OR child[tiab] OR youth[tiab] OR youths[tiab] | (validity[tiab] OR valid[tiab] OR reproducibility of results[MeSH] OR implementation[tiab] OR protocol[tiab] OR protocols[tiab] OR "best practices"[tiab] OR "best practice"[tiab] OR research design[MeSH]) OR  (discrimination[tiab] OR interpersonal relations[MeSH] OR socioeconomic factors[MeSH] OR disparity[tiab] OR disparities[tiab] OR health status disparities[MeSH] OR inequality[tiab] OR inequalities[tiab] OR equity[tiab] OR inequity[tiab] OR inequities[tiab] OR residence characteristics[MeSH] OR contrasting[tiab] OR "neighborhood"[tiab] OR "community characteristics"[tiab]) OR  (Synar[tiab] OR United States Food and Drug Administration[MeSH]) |
| 2015-05-11, remove "access" term because very broad and picking up many articles per Lara Handler consult, n = 522 | tobacco[MeSH] OR tobacco[tiab] OR tobacco use[MeSH] OR tobacco-use disorder[MeSH] OR tobacco products[MeSH] OR (smoking[MeSH] NOT marijuana smoking[MeSH]) OR smoking[tiab] OR cigarette[tiab] OR cigarettes[tiab] OR loosies[tiab] OR cigar[tiab] OR cigars[tiab] OR cigarillo[tiab] OR cigarillos[tiab] | sale[tiab] OR sales[tiab] OR retail[tiab] OR retailer[tiab] OR retailers[tiab] OR store[tiab] OR stores[tiab] OR sell[tiab] OR selling[tiab] OR shop[tiab] OR shops[tiab] OR tobacconist*[tiab] OR vending[tiab] OR vendor*[tiab] OR merchant*[tiab] OR "Commerce/legislation and jurisprudence"[MeSH] OR availability[tiab] | adolescen*[tiab] OR adolescent[MeSH] OR minors[tiab] OR minors[MeSH] OR underage[tiab] OR "under age"[tiab] OR student*[tiab] OR (young[tiab] near people[tiab]) OR children[tiab] OR juveniles[tiab] OR girls[tiab] OR boys[tiab] OR teenager*[tiab] OR teens[tiab] OR child[tiab] OR youth[tiab] OR youths[tiab] | (validity[tiab] OR valid[tiab] OR reproducibility of results[MeSH] OR implementation[tiab] OR protocol[tiab] OR protocols[tiab] OR "best practices"[tiab] OR "best practice"[tiab] OR research design[MeSH]) OR  (discrimination[tiab] OR interpersonal relations[MeSH] OR socioeconomic factors[MeSH] OR disparity[tiab] OR disparities[tiab] OR health status disparities[MeSH] OR inequality[tiab] OR inequalities[tiab] OR equity[tiab] OR inequity[tiab] OR inequities[tiab] OR residence characteristics[MeSH] OR contrasting[tiab] OR "neighborhood"[tiab] OR "community characteristics"[tiab]) OR  (Synar[tiab] OR United States Food and Drug Administration[MeSH]) |
| 2015-05-21, remove * for better comparability between search engines, n = 515 (none of the 7 removed are relevant) | tobacco[MeSH] OR tobacco[tiab] OR tobacco use[MeSH] OR tobacco-use disorder[MeSH] OR tobacco products[MeSH] OR (smoking[MeSH] NOT marijuana smoking[MeSH]) OR smoking[tiab] OR cigarette[tiab] OR cigarettes[tiab] OR loosies[tiab] OR cigar[tiab] OR cigars[tiab] OR cigarillo[tiab] OR cigarillos[tiab] | sale[tiab] OR sales[tiab] OR retail[tiab] OR retailer[tiab] OR retailers[tiab] OR store[tiab] OR stores[tiab] OR sell[tiab] OR selling[tiab] OR shop[tiab] OR shops[tiab] OR tobacconist[tiab] OR vending[tiab] OR vendor[tiab] OR vendors[tiab] OR merchant[tiab] OR merchants[tiab] OR "Commerce/legislation and jurisprudence"[MeSH] OR availability[tiab] | adolescence[tiab] OR adolescents[tiab] OR adolescent[tiab] OR adolescent[MeSH] OR minors[tiab] OR minors[MeSH] OR underage[tiab] OR "under age"[tiab] OR student[tiab] OR (young[tiab] near people[tiab]) OR children[tiab] OR juveniles[tiab] OR girls[tiab] OR boys[tiab] OR teenager[tiab] OR teenagers[tiab] OR teens[tiab] OR child[tiab] OR youth[tiab] OR youths[tiab] | (validity[tiab] OR valid[tiab] OR reproducibility of results[MeSH] OR implementation[tiab] OR protocol[tiab] OR protocols[tiab] OR "best practices"[tiab] OR "best practice"[tiab] OR research design[MeSH]) OR  (discrimination[tiab] OR interpersonal relations[MeSH] OR socioeconomic factors[MeSH] OR disparity[tiab] OR disparities[tiab] OR health status disparities[MeSH] OR inequality[tiab] OR inequalities[tiab] OR equity[tiab] OR inequity[tiab] OR inequities[tiab] OR residence characteristics[MeSH] OR contrasting[tiab] OR "neighborhood"[tiab] OR "community characteristics"[tiab]) OR  (Synar[tiab] OR United States Food and Drug Administration[MeSH]) |
| 2015-05-22, translated to PsycINFO | DE tobacco smoking OR DE nicotine OR  TI (tobacco OR tobacco OR smoking OR smoking OR cigarette OR cigarette OR cigarettes OR loosies OR cigar OR cigars OR cigarillo OR cigarillos) OR AB (tobacco OR tobacco OR smoking OR smoking OR cigarette OR cigarette OR cigarettes OR loosies OR cigar OR cigars OR cigarillo OR cigarillos) | DE (commerce) OR TI (sale OR sales OR retail OR retailer OR retailers OR store OR stores OR sell OR selling OR shop OR shops OR tobacconist OR vending OR vendor OR vendors OR merchant OR merchants OR OR availability) OR AB ( sale OR sales OR retail OR retailer OR retailers OR store OR stores OR sell OR selling OR shop OR shops OR tobacconist OR vending OR vendor OR vendors OR merchant OR merchants OR availability) | DE (Adolescent Development) OR TI (adolescence OR adolescents OR adolescent OR minors OR children OR juveniles OR girls OR boys OR teenager OR teenagers OR teens OR child OR youth OR youths OR "young people" OR underage OR "under age" OR student) OR AB (adolescence OR adolescents OR adolescent OR minors OR children OR juveniles OR girls OR boys OR teenager OR teenagers OR teens OR child OR youth OR youths OR "young people" OR underage OR "under age" OR student) | DE ("Consistency (Measurement)" OR Experimental Design OR Stereotyped Attitudes OR Socioeconomic Status OR Health Disparities OR Neighborhoods) OR TI (validity OR valid OR "reproducibility of results" OR implementation OR protocol OR protocols OR "best practices" OR "best practice" OR discrimination OR disparity OR disparities OR inequality OR inequalities OR equity OR inequity OR inequities OR contrasting OR neighborhood OR "community characteristics" OR Synar OR "Food and Drug Administration") OR AB (validity OR valid OR "reproducibility of results" OR implementation OR protocol OR protocols OR "best practices" OR "best practice" OR discrimination OR disparity OR disparities OR inequality OR inequalities OR equity OR inequity OR inequities OR contrasting OR neighborhood OR "community characteristics" OR Synar OR "Food and Drug Administration") |

TRANSLATION OF CONTROLLED VOCABULARY

| MeSH | PsycINFO |
| --- | --- |
| tobacco[MeSH] | nicotine |
| tobacco use[MeSH] | tobacco smoking |
| tobacco-use disorder[MeSH] | - |
| tobacco products[MeSH] | - |
| (smoking[MeSH] NOT marijuana smoking[MeSH]) | - |
| "Commerce/legislation and jurisprudence"[MeSH] | Commerce |
| adolescent[MeSH] | Adolescent Development |
| minors[MeSH] | - |
| reproducibility of results[MeSH] | Consistency (Measurement) |
| research design[MeSH] | Experimental Design |
| interpersonal relations[MeSH] | Stereotyped Attitudes |
| socioeconomic factors[MeSH] | Socioeconomic Status |
| health status disparities[MeSH] | Health Disparities |
| residence characteristics[MeSH] | Neighborhoods |
| United States Food and Drug Administration[MeSH] | - |

SEARCHES

2015-05-22, PubMed, N=515


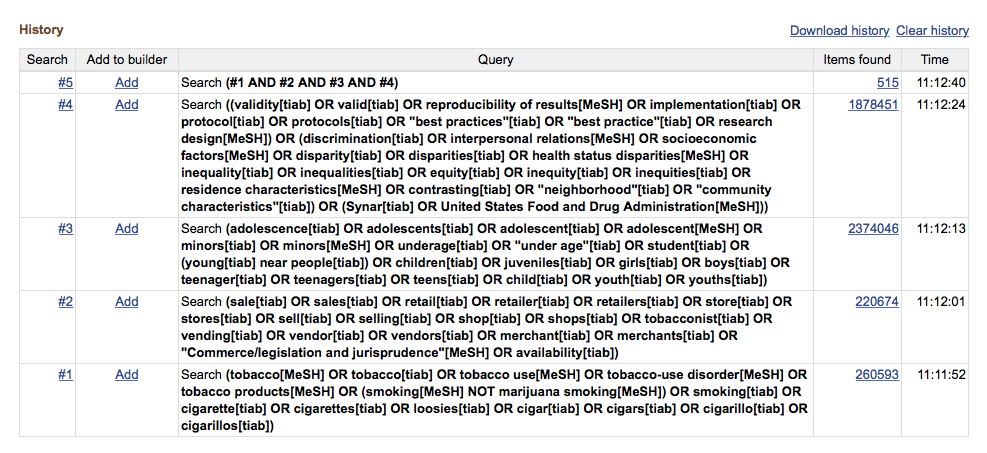


PsycINFO 2015-02-22, N=108


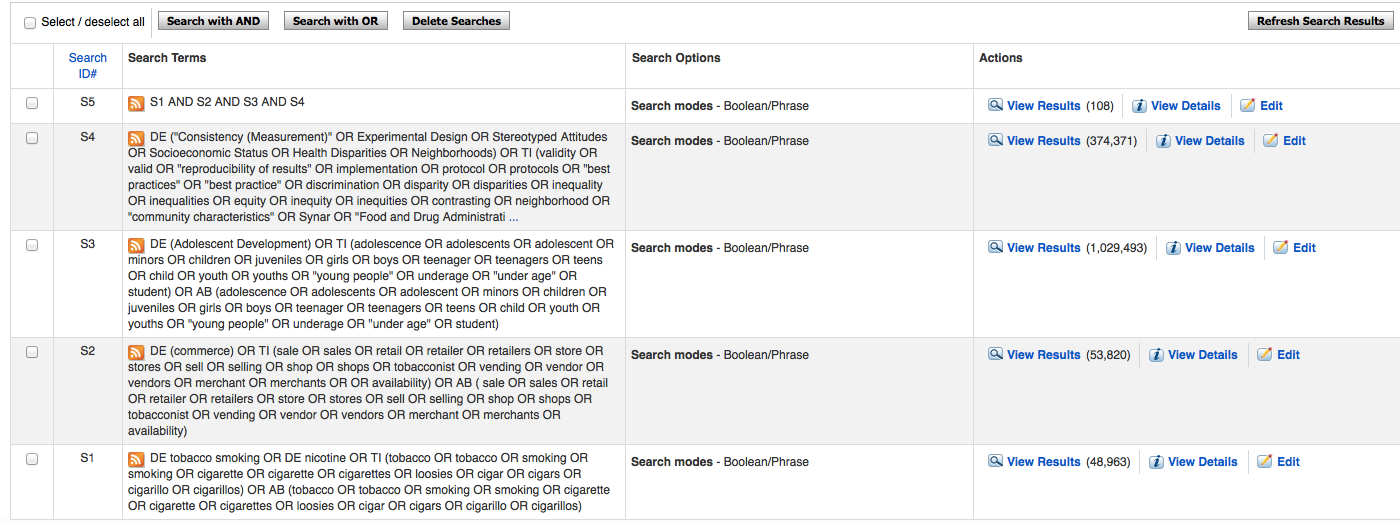


**References**

- **1.** Davis RM. Tobacco Control joins Index Medicus and Medline. *Tob Control.* Summer 1996;5(2):99. doi:10.1136/tc.5.2.99.
