# Supplementary material for: "May I Buy a Pack of Marlboros, Please?" A Systematic Review of Evidence to Improve the Validity and Impact of Youth Undercover Buy Inspections
Source: PLoS One. 2016 Apr 6;11(4):e0153152. doi: 10.1371/journal.pone.0153152 (PMC4822877; doi:10.1371/journal.pone.0153152)
Supplement: S1 Table — (DOCX) [file pone.0153152.s003.docx]

**S1 Table.** Evidence Regarding Youth, Protocol, and Neighborhood Correlates of Non-Compliance with Prohibition on Sales of Tobacco Products to Minors, May 22, 2015, N=44, Stratified by Risk of Confounding

| **Citation** | **Setting (n of checks), Year(s)** | **Sampling Strategy: Check Protocol** | **Age** | **Gender** | **Race/Ethnicity** | **Other Correlates; Risk of Bias** |
| --- | --- | --- | --- | --- | --- | --- |
| Asumda *et al*., 2009[1] (308) | FL (438), 2005 | Random: Synar protocol. No further detail provided. | - | - | - | Census block group proportion black negatively associated with sales, proportion Hispanic positively associated, and lower household income. In Miami, sales positively associated with proportion Hispanic, negatively with percent black, negative with income. In Tampa-St. Petersburg-Clearwater, NS. In Jacksonville, percent black negatively associated, percent Hispanic NS, income negatively associated. |
| Biglan *et al*., 1995[2] (578) | Four small communities, OR (1597), NR | NR: Youth age 15-17 in male-female pair, driven by adult. Pair enters store and alternates which person attempts buy. For girls, Marlboro or Camels were always requested. For boys, half of purchase attempts were for smokeless tobacco (Copenhagen or Kodiak). First get a food item or soft drink then ask for tobacco product. If sold, youth reported insufficient money and bought just the food item. If asked for ID, said they did not have it. If asked age, answer honestly. Instructed to make and keep eye contact. Multiple waves of data collection; each has new youth. | - | NS | - | To boys, smokeless tobacco more likely to be sold than cigarettes, FCG c = 4.01, df=1, p =0.045. |
| Clark *et al*., 2000[3] (180) | 36 states and DC (110,062), 1997-1999 | Census (but not achieved in all states): FDA inspection protocol (age 15-16 in year 1, added 17 in later years). Allowed to purchase gum or chips with tobacco product. Not allowed to alter appearance, but if normally wore beard or makeup allowed to keep doing so. Adult accompanied to store but not inside if it might give away the compliance check. | Older age associated with purchase, aOR 2.43 (2.31-2.59) for 17 vs. 15 years; aOR 1.52 (1.46-1.63) for 16 vs. 15. | Females more likely to be sold to than males, aOR 1.18 (1.13-1.23) | - | Purchase of smokeless tobacco, inspection after 5 p.m., Saturday inspection, suburban ZIP codes, and rural ZIP codes were associated with greater likelihood of sale. Adjusted for all other characteristics. |
| DiFranza *et al*., 1996[4] (571) | Massachusetts (480), NR | NR: Youths (1 male, 1 female for age 12, 13, 14, 15, 16, and 17) accompanied by research assistant who remained in car during purchase attempt. Instructed not to look older, ask for pack of cigarettes, state true age if asked, state cigarettes for themselves if asked, provide ID if asked. Each vender visited by three boys and three girls; each visit approximately 1 week apart. Order randomized. | Youths appearing age 16 and 17 were more likely to be sold to than 11-15 years of age, aOR 3.4 (2.0-5.8) | Girls more likely to be sold to than boys, OR 1.49 (1.01-2.19) | - | No age x gender interactions were significant. |
| DiFranza *et al*., 2001[5] (562) | Massachusetts (2013), FY1996-FY1997 | Census (within stratified clustered sample of neighborhoods): Youths (age 13-17) asked for cigarettes or selected from display, did not lie about age, did not use identification, did not attempt to persuade. | Youth age 13-15 were less likely to be sold to than youth age 16-17, aOR 0.36 (0.20-0.62) | - | - | Authors note that buy rates went from 4.2% for youth age 13 to 30.5% for youths age 16. |
| Erickson *et al*., 1993[6] (48) and Keay *et al*., 1993[7] (574) | Six sites (four neighborhood groups) in San Diego County, CA (294 baseline, 260 posttest), 1990 | Retailer passive consent (opt-out): Two-person of teen (age 12-17) and adult visited stores between 8 a.m. and 8 p.m. and attempted purchase (in two sites, if clerk rang purchase, buyer said they were "short on cash" -- others completed transaction). | Age significantly and positively associated with non-compliance at baseline (*x*^2^=41.20, p <0.001) and post-test (*x*^2^=12.85, p < 0.05). Purchases more likely for older youth. | Ericson: Girls more likely to successfully purchase than boys, *x*^2^=7.67, p <0.01, at baseline.  Keay: Female youth more likely to be sold to than males at baseline (*x*^2^=4.28, p≤0.05)  NS at post-test. | - | Differences in sales by community ethnicity. Lowest rates appear to be in black community; highest in white and Hispanic community. Chi-square results and N differ between reports, perhaps due to analysis of baseline using only complete data at post-test |
| Forester *et al*., 1992[8] (566) | Three suburban communities in Minneapolis-St. Paul metro area, MN (475), 1989 | Census: Youth (age 12-15) asked to purchase up to three times in one visit Marlboro cigarettes. Instructed to wear casual teen clothes; allowed to say they were 18 if asked. Three separate youth attempted (once each) at stores in April/May; then repeated with just a 14 year old boy and 15 year old girl in July. Two were smokers. | Older youths more likely to be sold to. | Girls more likely to be sold to than boys: "Even the youngest age group of girls was more successful purchasing over the counter than the oldest boys in this study" (p. 323). | - | - |
| Kirchner *et al*., 2015[9] (499) | Washington, DC (347), 2009-2010 | Random: Program staff and police officer remain outside while youth under 18 attempts purchase. | - | - | - | In block groups with >56% African-American residents, closer proximity to a high school or a park was associated with higher likelihood of a sale after control for store type, demographics, and advertising characteristics. |
| Klonoff *et al*., 1994[10] (374) | San Bernardino and Riverside County, CA (206), 1992 | Convenience: Stores located in middle and lower-middle class neighborhoods. Two white males aged 15 and 17 years old attempted to request a single cigarette alone. | - | - | - | Singles least likely to be sold in white neighborhoods, more likely in integrated neighborhoods, and most likely in minority neighborhoods (p. 619), *x*^2^=21.5, df=2, p < 0.001. |
| Klonoff *et al*., 1997[11] (171) and Landrine *et al*., 1998[12] (584) | San Bernardino County, CA (2592), 1993-1995 | NR: 2 (gender) x 3 (age: 10, 14, 16) x 3 (community: white, black, Latino) design with two youth per cell. Each youth attempted purchase at 72 stores alternating between a single cigarette and a pack of cigarettes | For packs, most significant predictor is age: 16 year olds vs. 14 year olds, x2=38.84, df=2, p = 0.00005  For singles, age is significant with a dose-response, x2, df=2 = 68.998. | For packs, girls more likely to be sold to, *x*^2^ = 14.72, df=1, p=0.0001.  For singles, gender not significant, *x*^2^_(df=1)_= 0.086. | Black (28.41%) versus white (21.06%) youth, x2 = 6.17, df=2, p < 0.05.  Black (28.41%) vs. Latino (17.36%), x2 = 14.95, df=2, p =0.0001  White vs. Latino, x2 = 1.96, df = 2, NS  For singles, ethnicity not significant, *x*^2^_(df=2)_ = 0.013 | For packs, no difference by neighborhood ethnicity, *x*2 = 2.30, df=2, p=0.32.  For singles, ethnicity of community differs by community, *x*^2^_(df=2)_ - 22.555 (more access in Black and Latino than white areas). |
| Landrine *et al*., 1997[13] (391) | San Bernardino County, CA (576), NR | Random: Stores selected in middle class communities. Youth aged 14 and 16 (equal number black, white, male, female, and by age) answered truthfully with extensive training to standardize approach. Research assistant stayed outside. Checks done between 3 p.m. and 7 p.m. Different youth attempted purchase at same stores over time, up to six different attempts: "May I have a pack of Camels, please?" | - | No significant difference, *x*^2^ = 0.068, df = 1, p = 0.79, n = 576. | Significant difference between black and white children, x2 = 5.366, df = 1, p = 0.02. Black children more likely to be sold to. | No significant difference by neighborhood race, black vs. white neighborhood, x2 = 0.031, df=1, p = 0.859; however, black children more likely to be sold cigarettes than white children in black neighborhoods but sold same amount of cigarettes in white neighborhoods. |
| Landrine *et al.*, 2000[14] (309) | San Bernardino County, CA (2664), 1994-1999 at five time points | Random: Stores selected in black, white, and Latino communities. Minors matched by size and appearance (children of college faculty). Checks done between 3 p.m. and 7 p.m. Youth asked, “May I buy a pack of Marlboros, please?” (p. ii16). | - | - | - | No differences in change over time by neighborhood characteristics. |
| Landrine *et al*., 2000[15] (560) | San Bernardino County, CA (828), 1998 | Random: Checks done between 3 p.m. and 7 p.m. using age 16 youth (6 girls, 6 boys, 2 black, 2 white, 2 Latino). Each store visited by each minors over a 3-month period, no more than 2 per week. "May I have a pack of Marlboros, please?" | - | Gender NS, x2 = 0.006, df=1 | Higher sales to black and Latino than white youth, x2 = 12.61, df=2, p=0.002. Remained significant in logistic regression model controlling for youth, neighborhood, and clerk characteristics: Minority youths were 2.5x more likely than white youths to be sold a tobacco product. | Neighborhood ethnicity NS, *x*^2^ = 1.962, df=2 |
| Landrine *et al*., 2010[16] (162) | California (3361), 1999-2003 | Random: Youth age 15-16 attempted purchase without an identification card and answering questions truthfully. | 16 year olds more likely to be sold to than 15 year olds: OR: 1.74, 1.39-2.19 | Girls more likely to be sold to than boys (controlling for age), OR: 1.61, 1.30-1.99 | Black youths more likely to be sold to than white youths (controlling for age, gender, and clerk ethnicity), OR: 1.31, 1.00-1.72 (p=0.047) | Gender x ethnicity, age x ethnicity interactions significant |
| Lipperman-Kreda *et al*., 2014[17] (85) | 50 California Cities (997), 2011 | Randomly: Purchasers judged to look under 18 but were over 18, did not lie about age, purchased alternating Newport and Marlboro cigarettes, and did not provide ID. | Buyer age associated with non-compliance in adjusted model: OR 1.66 (1.17-2.38) | NS adjusted for city, retailer, buyer, and clerk characteristics | - | Descriptive statistics suggest more non-compliance with Newport purchase than Marlboro |
| Lipton *et al*., 2008[18] (568) | Los Angeles, CA (689), 2001 | Stratified Random Sample: Youth age 15-16 were matched to communities (black youth in neighborhoods with more black residents). |  |  |  | At ZIP code level using regression the following were related to noncompliance: population density, median household income, percent foreign born, percent black, and percent 15-24 age. Percent poverty NS. All adjusted for each other. |
| Pearson *et al*., 2007[19] (561) | King County, WA (8879), 2001-2005 | Randomly: Youth aged 14-17, nonsmoking, not lie about age. | Age 17 vs. 14: OR 7.81, 2.69-22.62 (adjusting for store, youth, year, and neighborhood characteristics) | Compared to male youth with male clerk, a female/female combination had higher sales rates as did a male youth/female clerk combination (adjusting for store, youth, year, and neighborhood characteristics). | - | Sales lowest on Mondays; When ID requested but not shown, sales decreased to very low rates (lower than when ID shown). Sales higher from vending machine. Time of day and neighborhood poverty, NS, but some evidence of higher sales as time increases |
| *Schmidt, 2001[20] (129) | Baltimore, MD (237), 2000 | Racially concordant pairs of girls, age 16.5-17.5, entered a store together. One of the pair attempted to purchase cigarettes in alternating fashion. All were non-smokers. White girls asked for "a pack of Marlboro Reds" and Black girls for "a pack of Newports." Answered questions about age truthfully, showed real id if asked. | - | - | Differences by sale rate when discordant race of minor and clerk, *x*^2^, df=1, n=237, = 4.67.  Black girls more likely to be sold to than white, *x*^2^, df=1, n=237 = 11.12. Remains significant after control for clerk, store, and neighborhood characteristics. | Mean family income lower in neighborhoods where purchases were successful, *x*^2^, df=1, n=236 = 9.49. Remains significant after control for clerk, store, and % Black variables.  Percentage of Black residents associated with successful purchase, *x*^2^, df=1, n=236 = 13.13 |
| Widome *et al*., 2012[21] (405) | Midwestern metropolitan area (467), NR | Census: No standard script, age 16-17, using real ID. | - | - | - | Stores in census block groups with greater proportion of Hispanic residents more likely to fail compliance check, *p* = 0.045. Other demographic characteristics NS |
| *In the studies below, we were unable to rule out the possibility of confounding between the age, gender, race, or assignment area of the youth* | | | | | | |
| Altman *et al*., 1989[22] (573) | Santa Clara County, CA (412), 1988 | Convenience: Adults remained in car while minor attempted purchase of Marlboro cigarettes. Minors answered age questions honestly and, if asked, reported cigarettes were "for me." | Significantly fewer 14 than 15 or 16 year olds were able to purchase. | More girls than boys were able to purchase, p = 0.02 (in follow-up test). | - | The text and table offer conflicting information about the significance of these findings.  Unclear of age x gender distribution of minors. |
| Arday *et al*., 1997[23] (558) | Austin, TX (165), 1993 | Random: Adults entered store 30s to 1 min. before minors, gave actual ages if asked and said product was for themselves. Purchases not completed for legal reasons; successful purchase defined as transaction being rung up on register. Minor then noted they did not have enough money. | NS | NS | NS | Prior experience, NS. Rural areas had lower sale rate.  Youth were assigned to a region and thus youth characteristic associations may be confounded by region. |
| Bridges *et al*., 1996[24] (572) | Broward County, FL (125), 1996 | Random: Minor (age 15-17) and adult attempted purchase. Adult entered store first and minor then entered and attempted purchase of smokeless tobacco product. Purchase was not completed (minor reported insufficient money). | NS | NS | - | Two minors were female (age 15) and three was male (age 15, 16, 17); age and gender are confounded in this study. |
| Bridges *et al*., 1995[25] (565) | Palm Beach County, FL (88), 1994 | Random: Minor (age 11-17) and adult attempted purchase. Adult entered store first and minor then entered and attempted purchase of smokeless tobacco product. Purchase was not completed (minor reported insufficient money). | Greatest likelihood of sale was to 17 year old female. | Greatest likelihood of sale was to 17 year old female. | - | Three minors were female (age 11, 14, and 17) and one was male (age 14); age and gender are confounded in this study. |
| Cismoski & Sheridan, 1993[26] (587) | Fon du Lac, Wisconsin (67), 1993 | Census: Two boys and two girls, age 14 attempt to purchase at vending machine if available or over the counter (results reported here are for over the counter). | - | No difference by gender | - | With only four youth, it is unclear if other youth characteristics may be a confound. |
| Cohen *et al*., 1995[27] (590) | 10 North Carolina Counties (1009), 1994 | Convenience: Minors age 11-17 trained and with adult chaperone attempted purchase. No attempt to recruit youth who looked older than their age. | Significantly higher rate of sales for ages 16/17 than for age 15 and under. | In model adjusted for location of cigarettes, age questioned, sign posted, and store type, boys were less likely than girls to be sold to, OR 0.25 (0.12-0.51). |  |  |
| *DiFranza, Savageau, and Bouchard, 2001[28] (269) | Eight communities, Massachusetts (320), NR | Two attempts made on same day (within 30 minutes to standardize clerk) at 160 retailers, by pairs of youth matched age, sex, and race/ethnicity. Study compares two types of protocols. | There is a dose response with higher age being associated with greater likelihood of purchase. | NS | - | Authors note study purpose was not to compare minor characteristics. One pair of youth conducted all inspections within a given community. Cannot rule out confounding. |
| Glanz *et al*., 2007[29] (397) | Hawaii (203), 2003 | Random: Age 17-17, two-thirds female, race/ethnicity representative of state. Did not carry identification and should report true age. Adult waited outside. | "more sales to older youths" (p. 57) | "more sales to males" (p. 57) | - | Adjusted reported come from a single year and unadjusted results are not reported; study may be underpowered for the number of variables in the logistic model with an N of 203.  Youth were assigned to a region and thus youth characteristic associations may be confounded by region. |
| Jason *et al*., 1996[30] (580) | Woodbridge, IL (480), 1991-1994 | Minors age 12-17 were accompanied by a plainclothes police officer. Gender and race not reported. | When older minors were used, purchase rates were generally higher. | - | - | It is not possible to distinguish any secular trend from the results; different minors were used at different time points. |
| *Jason *et al*., 1996[31] (569) | Chicago, IL (120 stores, assessed repeatedly across time), 1993-1995 | NR: Diverse minors appearing age 16-17 of both genders entered store along with adult outside and asked to purchase Marlboro or Camel light cigarettes. If asked, minor stated he or she had no ID. If asked, minor told actual age. Training included role-plays. Stores (n=99 at final wave) stratified by community ethnicity and assigned to enforcement at no (control), 2-, 4-, and 6-month intervals. | - | More sales to female minors at baseline, *x*^2^_(df=1, n=154)_ = 30.86; NS during intervention phase. | NS | NS for differences by neighborhood ethnicity.  Cannot rule out age, gender, race, and neighborhood confounding. |
| *Klonoff & Landrine, 2004[32] (354) | San Bernardino and Riverside County, CA (1,600), NR | : 1,600 purchase attempts in 232 stores with extensive training and seven waves of data collection, alternating standard protocol with alternative protocols to assess for secular trends. Each wave was separated by 4 to 6 weeks. Checks took place between 3 p.m. and 7 p.m. on weekdays and 9 a.m. and 4 p.m. on weekends. Racially/ethnically diverse participants, aged 15 - 17 (n=21). All were non-smokers. | "Youth age affected sales as well, with 16-year-olds 3.7 times more likely and 17-year olds 2 times more likely than 15-year-olds to be sold cigarettes." (p. 519). | NS | NS | Age, ethnicity, and gender may be confounded. |
| *Landrine & Klonoff, 2001[33] (581) | California Cities (n=22) (674), NR | Random: Non-smoking minors age 15-17 attempted purchase at three time points to assess secular trends using variations on a protocol to test effects of flashing an ID card. | Age 17 vs. age 15: OR 10 (3.62-28.15)  Age 15 vs. age 15: OR 3.5 (1.53-8.28) | - | - | Cannot rule out confounding based on information presented. |
| *Landrine & Klonoff, 2003[34] (536) | San Bernardino and Riverside County, CA (915), NR | Random: Based on interviews with youth smokers, researchers designed a alternative protocol that was implemented by non-smoking youth age 15-17 in 232 randomly-selected tobacco retailers. Retailers visited no more than 7 youths per week (1 per day and 1 per clerk). Additional waves of data collection using the standard protocol (1 prior, 2 after) were used to assess secular trends. | Age 17 vs. age 15, 8 times more likely to be sold tobacco.  Age 16 vs. age 15, 3.5 times more likely to be sold tobacco. | - | Hispanic/Latino youth 5 times more likely to be sold tobacco than White youths. | Cannot rule out confounding based on information presented |
| *Levinson, Hendershott, and Byers, 2002[35] (236) | Six urban and suburban counties, CO (1083), 2001 | Non-smoking youths age 14-17 (n=12, 10 were male), most with previous compliance check experience, purchase attempts varying the presentation or non-presentation of a Colorado state ID (which notes the holder is under age 21). Males were clean shaven. Females wore no makeup. Adult supervisors were present in the retailer during the check. | Dose response with age from 1.2% sales rate for age 14 to 12.6% sales rate for age 17. | Higher sales rate for males than females (8.2% vs. 0.8%) | - | Cannot rule out confounding based on information presented. |
| *Levinson & Patnaik, 2013[36] (353) | Three counties, CO (1079), 2011-2012 | Census: Using standard Synar protocol in one urban, one suburban, and one resort county, researchers conducted test-retest reliability assessment in a census of 671 tobacco retailers. Minors (four female, seven male) were age 15.5-16, with A/B grades, non-smokers, clean shaven (males) with no tattoos, makeup, and modest/casual dress.  Could enter alone or with another minor, answer questions evasively, buy snack or drink, use ID or not, and visit store several times before attempted purchase. Adults could stay outside or enter. | NS | NS | - | Cannot rule out confounding based on information presented. |
| Ma *et al*., 2001[37] (138) | Philadelphia, PA (1649), 1994-1998 | Randomly, Reported, or Convenience: Reported merchants sent letter noting compliant received. Youth ages 14 to 17, accompanied by adult, not lie about age, and show real ID | Age 17 vs. 14: OR 2.79, 1.67-4.67  Age 17 vs. 15: OR 2.16, 1.52-3.08  Age 17 vs. 16: OR 2.64, 1.82-3.84 | Females vs. males, OR 1.38, 1.09-1.75 | - | Brand of cigarettes report as significant predictor in abstract, but not otherwise discussed.  Breakdown of age x gender of youth is not reported. |
| Mead, 1993[38] (588) | Green Bay, WI (66), 1992 | NR: NR. Two males age 15-16; three females, aged 13. Adults witnessed sales; some were video-recorded. | - | No difference by gender. | - | Age and gender may be confounded. |
| Missouri Coalition on Smoking and Health *et al*., 1993[39] (563) | Five Central Missouri Towns (89), MO, 1992; Austin, TX, metro area (94), 1993 | NR: Minor (age 13-14 in MO and 14-17 in TX) and adult attempted purchase. Adult entered store first and minor then entered and attempted purchase of cigarettes (MO) or smokeless (TX). Purchase was not completed (minor reported insufficient money). | - | NS, sales more likely for female buyers, p = 0.1 in MO.  NS, p =.0 in TX | - | Age and gender are confounded in this study. |
| Radecki *et al*., 1993[40] (567) | 93 U.S. communities, 37 states (2337 including those in 4 Canadian cities), 1991-1993 | NR: Youth age 16 (in U.S.) entered store, requested cigarettes, paid, and left; said they were 18 if asked. One was a smoker. | - | U.S. results NS | - | Certain youth used only in certain cities. |
| Ravesloot *et al*., 1990[41] (564) | Denver, CO (121), 1989 | Random: Adult and minor (age 9-17) attempted but did not complete purchases | NS (compared above and below age 14) | NS | - | Purchase attempts more successful in rural towns around Denver than in suburban Denver.  Age x gender breakdown not reported. |
| Skretny *et al*., 1990[42] (575) | Erie County, NY (120), NR | NR: Minors age 14-16 visited stores and asked to purchase Marlboro Lights. If asked, they stated actual age and that cigarettes for for their own use. | NS | NS | - | Seven participating minors (3 age 14, 2 age 15, 2 age 16; gender breakdown not reported. |
| Voorhees *et al*., 1997[43] (67) | East Baltimore catchment area of Johns Hopkins Medical Institution (83), NR | Random: Pairs of 14-16 year old minors (black males, black females, white females; no white males) visited stores in racially concordant area as a pair and alternated purchase attempts (1 per store) | NS | - | Racial discordance of clerk and buyer identified as significant predictor, OR: 8.18, 1.86-36.00, controlling for other variables (not reported). |  |
| Williams *et al*., 2014[44] (519) | Hawai'i (except islands of Moloka'i and Lana'i) (174), 2013 | Random: Youth age 15-17 attempted purchase, telling truth about age if asked, did not use identification cards, sometimes purchased other items (e.g., gum or drink). Minors reflected race/ethnicity of state. | More sales to older minors (some but not all years) | More sales to males (some but not all years) | - | Study may be underpowered for the number of variables in the logistic model with an N of 174.  Youth were assigned to a region and thus youth characteristic associations may be confounded by region. |
| Xaverius *et al*., 1996[45] (589) | Chicago, IL (60), 1994 | Randomly selected stores divided into "White, Hispanic, and African-American ethnic geographical areas" (p. 69). Two 16-year-old males (one White, one Puerto Rican) attempted smokeless tobacco purchases. Reported true age if asked and did not provide id. | - | - | - | Significant difference in sales by community ethnicity, sales rate of 100% in African-American area, 90% in White area, 70% in Latino area, *x*^2^, df=2 = 8.08.  Because there are only two minors, it is not possible to rule out confounding of other minor characteristics. |

* denotes that paper is also an experiment and is also in Evidence Table A.

**References**

1. Asumda F, Jordan L. Minority youth access to tobacco: a neighborhood analysis of underage tobacco sales. Health Place. 2009;15(1):140-7. Epub 2008/05/17. doi: 10.1016/j.healthplace.2008.03.006. PubMed PMID: 18482856.

2. Biglan A, Henderson J, Humphrey D, Yasui M, Whisman R, Black C, et al. Mobilising positive reinforcement to reduce youth access to tobacco. Tob Control. 1995;4(1):42-8. doi: 10.1136/tc.4.1.42.

3. Clark PI, Natanblut SL, Schmitt CL, Wolters C, Iachan R. Factors associated with tobacco sales to minors: lessons learned from the FDA compliance checks. JAMA. 2000;284(6):729-34. Epub 2000/08/06. PubMed PMID: 10927782.

4. DiFranza JR, Savageau JA, Aisquith BF. Youth access to tobacco: the effects of age, gender, vending machine locks, and "it's the law" programs. Am J Public Health. 1996;86(2):221-4. Epub 1996/02/01. PubMed PMID: 8633739; PubMed Central PMCID: PMCPMC1380331.

5. DiFranza JR, Celebucki CC, Mowery PD. Measuring statewide merchant compliance with tobacco minimum age laws: the Massachusetts experience. Am J Public Health. 2001;91(7):1124-5. Epub 2001/07/10. PubMed PMID: 11441743; PubMed Central PMCID: PMCPMC1446711.

6. Erickson AD, Woodruff SI, Wildey MB, Kenney E. A baseline assessment of cigarette sales to minors in San Diego, California. J Community Health. 1993;18(4):213-24. Epub 1993/08/01. PubMed PMID: 8408751.

7. Keay KD, Woodruff SI, Wildey MB, Kenney EM. Effect of a retailer intervention on cigarette sales to minors in San Deigo County, California. Tob Control. 1993;2(2):145-51. doi: 10.1136/tc.2.2.145.

8. Forster JL, Hourigan M, McGovern P. Availability of cigarettes to underage youth in three communities. Prev Med. 1992;21(3):320-8. Epub 1992/05/01. PubMed PMID: 1614994.

9. Kirchner TR, Villanti AC, Cantrell J, Anesetti-Rothermel A, Ganz O, Conway KP, et al. Tobacco retail outlet advertising practices and proximity to schools, parks and public housing affect Synar underage sales violations in Washington, DC. Tob Control. 2015;24(e1):e52-8. Epub 2014/02/27. doi: 10.1136/tobaccocontrol-2013-051239. PubMed PMID: 24570101.

10. Klonoff EA, Fritz JM, Landrine H, Riddle RW, Tully-Payne L. The problem and sociocultural context of single-cigarette sales. Jama. 1994;271(8):618-20. Epub 1994/02/23. PubMed PMID: 8301795.

11. Klonoff EA, Landrine H, Alcaraz R. An experimental analysis of sociocultural variables in sales of cigarettes to minors. Am J Public Health. 1997;87(5):823-6. Epub 1997/05/01. PubMed PMID: 9184513; PubMed Central PMCID: PMCPmc1381057.

12. Landrine H, Klonoff EA, Alcaraz R. Minors' access to single cigarettes in California. Prev Med. 1998;27(4):503-5. Epub 1998/07/22. doi: 10.1006/pmed.1998.0326. PubMed PMID: 9672942.

13. Landrine H, Klonoff EA, Alcaraz R. Racial discrimination in minor's access to tobacco. Journal of Black Psychology. 1997;23(2):135-47. doi: 10.1177/00957984970232004. PubMed PMID: 1997-04329-003.

14. Landrine H, Klonoff EA, Reina-Patton A. Minors' access to tobacco before and after the California STAKE Act. Tob Control. 2000;9 Suppl 2:II15-7. Epub 2000/06/07. PubMed PMID: 10841587; PubMed Central PMCID: PMCPMC1766282.

15. Landrine H, Klonoff EA, Campbell R, Reina-Patton A. Sociocultural variables in youth access to tobacco: replication 5 years later. Prev Med. 2000;30(5):433-7. Epub 2000/06/14. doi: 10.1006/pmed.2000.0652. PubMed PMID: 10845753.

16. Landrine H, Corral I, Klonoff EA, Jensen J, Kashima K, Hickman N, et al. Ethnic disparities in youth access to tobacco: California statewide results, 1999-2003. Health Promotion Practice. 2010;11(1):132-9. doi: 10.1177/1524839908317230. PubMed PMID: 2010-00004-019.

17. Lipperman-Kreda S, Grube JW, Friend KB. Contextual and community factors associated with youth access to cigarettes through commercial sources. Tobacco Control: An International Journal. 2014;23(1):39-44. doi: 10.1136/tobaccocontrol-2012-050473. PubMed PMID: 2014-03752-005.

18. Lipton R, Banerjee A, Levy D, Manzanilla N, Cochrane M. The spatial distribution of underage tobacco sales in Los Angeles. Subst Use Misuse. 2008;43(11):1594-614. Epub 2008/08/30. doi: 10.1080/10826080802241110. PubMed PMID: 18752162.

19. Pearson DC, Song L, Valdez RB, Angulo AS. Youth tobacco sales in a metropolitan county: Factors associated with compliance. American Journal of Preventive Medicine. 2007;33(2):91-7. doi: 10.1016/j.amepre.2007.04.010.

20. Schmitt CL. The effect of decision heuristics and ethnicity on cigarette sales to minor girls. Baltimore, MD: University of Maryland, Baltimore County; 2001.

21. Widome R, Brock B, Noble P, Forster JL. The relationship of point-of-sale tobacco advertising and neighborhood characteristics to underage sales of tobacco. Evaluation & the Health Professions. 2012;35(3):331-45. doi: 10.1177/0163278712447624. PubMed PMID: 2012-21477-005.

22. Altman DG, Foster V, Rasenick-Douss L, Tye JB. Reducing the illegal sale of cigarettes to minors. JAMA. 1989;261(1):80-3. Epub 1989/01/06. PubMed PMID: 2908999.

23. Arday DR, Klevens RM, Nelson DE, Huang P, Giovino GA, Mowery P. Predictors of tobacco sales to minors. Preventive Medicine. 1997;26:8-13. doi: 10.1006/pmed.1996.9984.

24. Bridges FS, Welsh RL, Malecki JM. Accessibility to minors of smokeless tobacco products--Broward County, Florida, March-June 1996. MMWR Morb Mortal Wkly Rep. 1996;45(49):1079-82. Epub 1996/12/13. PubMed PMID: 8975122.

25. Bridges FS, Malecki JM. Minors' access to smokeless tobacco -- Florida, 1994. MMWR Morb Mortal Wkly Rep. 1995;44(44):839-41. doi: <http://www.cdc.gov/mmwr/preview/mmwrhtml/00039511.htm>.

26. Cismoski J, Sheridan M. Availability of cigarettes to under-age youth in Fond du Lac, Wisconsin. Wis Med J. 1993;92(11):626-30. Epub 1993/11/01. PubMed PMID: 8303900.

27. Cohen JE, Stanley LC, Martin JD, Goldstein AO. Illegal sales of cigarettes to minors in North Carolina. N C Med J. 1995;56(1):59-63. Epub 1995/01/01. PubMed PMID: 7862209.

28. DiFranza JR, Savageau JA, Bouchard J. Is the standard compliance check protocol a valid measure of the accessibility of tobacco to underage smokers? Tob Control. 2001;10(3):227-32. Epub 2001/09/07. PubMed PMID: 11544386; PubMed Central PMCID: PMCPMC1747587.

29. Glanz K, Jarrette AD, Wilson EA, O'Riordan DL, Jacob Arriola KR. Reducing minors' access to tobacco: eight years' experience in Hawaii. Prev Med. 2007;44(1):55-8. Epub 2006/11/07. doi: 10.1016/j.ypmed.2006.08.021. PubMed PMID: 17084888.

30. Jason LA, Billows WD, Schnopp-Wyatt DL, King C. Long-term findings from Woodridge in reducing illegal cigarette sales to older minors. Eval Health Prof. 1996;19(1):3-13. Epub 1996/02/07. PubMed PMID: 10186901.

31. Jason L, Billows W, Schnopp-Wyatt D, King C. Reducing the illegal sales of cigarettes to minors: analysis of alternative enforcement schedules. J Appl Behav Anal. 1996;29(3):333-44. Epub 1996/01/01. doi: 10.1901/jaba.1996.29-333. PubMed PMID: 8926225; PubMed Central PMCID: PMCPMC1283996.

32. Klonoff EA, Landrine H. Predicting Youth Access to Tobacco: The Role of Youth Versus Store-Clerk Behavior and Issues of Ecological Validity. Health Psychol. 2004;23(5):517-24. doi: 10.1037/0278-6133.23.5.517. PubMed PMID: 2004-18051-009.

33. Landrine H, Klonoff EA, Lang D, Alcaraz R. Use of identification cards by underage youth to purchase tobacco. JAMA. 2001;285(18):2329. Epub 2001/05/10. PubMed PMID: 11343479.

34. Landrine H, Klonoff EA. Validity of Assessments of Youth Access to Tobacco: The Familiarity Effect. American Journal of Public Health. 2003;93(11):1883-6. doi: 10.2105/AJPH.93.11.1883. PubMed PMID: 2003-09633-012.

35. Levinson AH, Hendershott S, Byers TE. The ID effect on youth access to cigarettes. Tob Control. 2002;11(4):296-9. Epub 2002/11/15. PubMed PMID: 12432154; PubMed Central PMCID: PMCPMC1747672.

36. Levinson AH, Patnaik JL. A practical way to estimate retail tobacco sales violation rates more accurately. Nicotine & Tobacco Research. 2013;15(11):1952-5. doi: 10.1093/ntr/ntt084. PubMed PMID: 2013-35572-021.

37. Ma GX, Shive S, Tracy M. The effects of licensing and inspection enforcement to reduce tobacco sales to minors in greater Philadelphia, 1994-1998. Addictive Behaviors. 2001;26(5):677-87. doi: 10.1016/S0306-4603(00)00150-7. PubMed PMID: 2002-06536-006.

38. Mead R. Teen access to cigarettes in Green Bay, Wisconsin. Wis Med J. 1993;92(1):23-5. Epub 1993/01/01. PubMed PMID: 8424278.

39. Missouri Coalition on Smoking and Health, Romeis JC, Brownson RC, Davis JR, Cooperstock LR, Huang PP, et al. Minors' access to tobacco--Missouri, 1992, and Texas, 1993. MMWR Morb Mortal Wkly Rep. 1993;42(7):125-8. Epub 1993/02/26. PubMed PMID: 8437540.

40. Radecki TE, Zdunich CD. Tobacco sales to minors in 97 US and Canadian communities. Tob Control. 1993;2(4):300-5. doi: 10.1136/tc.2.4.300.

41. Ravesloot L, Young WF, Walkington DA. From the Centers for Disease Control. Cigarette sales to minors--Colorado, 1989. JAMA. 1990;264(21):2734. Epub 1990/12/05. PubMed PMID: 2232053.

42. Skretny MT, Cummings KM, Sciandra R, Marshall J. An intervention to reduce the sale of cigarettes to minors. N Y State J Med. 1990;90(2):54-5. Epub 1990/02/01. PubMed PMID: 2304661.

43. Voorhees CC, Swank RT, Stillman FA, Harris DX, Watson HW, Jr., Becker DM. Cigarette sales to African-American and white minors in low-income areas of Baltimore. Am J Public Health. 1997;87(4):652-4. Epub 1997/04/01. PubMed PMID: 9146447; PubMed Central PMCID: PMCPMC1380848.

44. Williams RJ, Kobayashi L, Fujimoto T, Swartz K, Whitehead TK, Bonifacio N. Trends in underage tobacco sales: an update on the past decade of compliance checks in Hawai'i. Hawaii J Med Public Health. 2014;73(10):315-21. Epub 2014/10/23. PubMed PMID: 25337449; PubMed Central PMCID: PMCPMC4203452.

45. Xaverius PK, Billows WD, Jason LA, King C. Research on the sale of smokeless tobacco to adolescents. Tob Control. 1996;5(1):69-70. Epub 1996/01/01. PubMed PMID: 8795864; PubMed Central PMCID: PMCPMC1759493.
